# Intramuscular Degloving Injury of the Rectus Femoris From Kickball: A Case Report and Review

**DOI:** 10.7759/cureus.42230

**Published:** 2023-07-21

**Authors:** Leo Meller, Michael C Oca, Katherine Wilson, Matthew Allen, Edward Smitaman, Sandhya Kalavacherla, Kenneth Vitale

**Affiliations:** 1 Department of Orthopedic Surgery, Division of Sports Medicine, University of California San Diego School of Medicine, La Jolla, USA; 2 Department of Radiology, University of California San Diego School of Medicine, La Jolla, USA

**Keywords:** recreational sports, return to sport, rectus femoris, athletic injuries, orthopedic sports medicine, degloving injury, quadriceps muscle

## Abstract

Intramuscular degloving injuries (IDIs) are a rare and unique type of muscle injury where there is a dissociation between the inner and outer components of a particular muscle. This type of injury is seen exclusively within the rectus femoris (RF) muscle due to its unique muscle-within-a-muscle anatomy and represents 9% of RF injuries. Despite the significance of this injury, limited knowledge exists regarding the mechanism, management, and prognosis of IDIs, and IDIs are not currently included among the various muscle injury classifications. We present a 38-year-old active male with a one-week history of acute onset right anterior mid-thigh pain and palpable lump after playing kickball. Right thigh MRI revealed an IDI of the RF muscle, edema within the inner and outer muscular portions of the muscle, and a retraction of the torn inner indirect myotendinous complex of the RF. He was managed with physical therapy while being advised to avoid aggressive quadriceps contractions, high-intensity, or high-impact exercise. This is the first reported case of an IDI that occurred in an older recreational athlete (versus young competitive athletes), and the first case of an IDI in a kicking sport other than soccer (kickball). This case emphasizes the importance of a broader awareness of this injury, and a heightened index of suspicion is advised in assessing potential IDIs to improve patient prognosis and rehabilitation. Given the limited understanding and rarity of this injury, we also provide a comprehensive review describing the IDI to the RF.

## Introduction

The rectus femoris (RF) is the most injured quadriceps muscle [1-2] and is frequently affected in both athletic and non-athletic populations. The incidence of RF injuries depends on a myriad of factors, including type of sport, level of competition, and sex, with a higher incidence in male athletes [3]. As a biarticular muscle located in the anterior thigh, the RF is uniquely designed to handle high shortening velocities and significant length changes that are involved in dynamic movements such as kicking and sprinting [4]. As a result, the most common mechanisms of injury stem from activities that involve kicking, knee extension, forced hip extension, and sprinting [5]. To understand the unique pathophysiology of Intramuscular degloving injuries (IDIs), it is important to delve deeper into the muscle’s intricate architecture. The muscle’s dual-headed origin, with the direct head stemming from the anterior inferior iliac spine (AIIS) and the indirect head from the superior acetabular ridge, contributes to its complex structure. The two heads merge to form the conjoined tendon, with the indirect head contributing to the deep intramuscular component [4]. This intricate architecture contributes its unique muscle-within-a-muscle configuration and predisposes the RF to a rare type of injury known as the IDI.

IDIs are uniquely characterized by the dissociation and potential retraction of the inner bipennate muscle from the outer unipennate muscle, which occurs in the absence of involvement of the myofascial or myotendinous junctions, or the intratendinous regions. Furthermore, the separation of these two muscles of the RF contributes to the *finger-in-a-glove* appearance characteristically seen in MRI [6]. Although IDIs account for only approximately 9% of RF injuries, they are clinically significant due to their ability to significantly impair mobility and function [7]. A potential complication in degloving injuries occurs when fluid accumulates between the inner and outer muscular components, which can lead to infection or necrosis and put the skin at risk for ulceration and ischemia [8]. Furthermore, the retraction of the indirect head of the muscle in such injuries contributes to loss of strength, decreased mobility, chronic pain, and stiffness [9].

While IDIs confer significant morbidity, there is a paucity of literature on these injuries. Reports on recovery and return to play can vary widely, with recovery times reportedly ranging from a few weeks to several months [6-7,10]. Moreover, while previous research has described the anatomy of the RF, injuries to this muscle may not always fit into the traditional grading system for muscle injuries [2]. The traditional grading scale for muscle injuries consists of three grades: grade 1 for mild strain, grade 2 for partial tears, and grade 3 for complete tears. IDIs do not fit well into any of these categories. Studies have shown that while IDIs may appear similar to grade 3 injuries on MRI, other issues, including differences in radiological grading scales (such as different MRI classification systems) and clinical factors (such as average return to play interval), contribute to clinicians categorizing this injury more closely to a grade 2 muscle lesion [6,11-12].

Hence, there is a significant gap in the literature regarding IDIs. Given the rarity and thus limited reports of IDI, more detailed patient-level data, including demographics, presenting symptoms, cause of injury, physical examination findings, prognosis, and outcome is warranted. In addition, a comprehensive review of the existing literature on IDI is necessary to enhance providers’ understanding of this important yet often overlooked injury. In this paper, we report the first case of an IDI to the RF occurring in an older recreational athlete (all previous cases aged 15-22 years) from playing kickball and provide a comprehensive narrative review of the current literature on IDI.

## Case presentation

A 38-year-old active male with no relevant past medical history presented with a one-week history of acute-onset, right anterior-middle thigh pain, and palpable lump after playing kickball in 2022. On a numeric pain rating scale, his pain level was rated as 6/10. The pain was continuous, exacerbated by activity and exercise, and relieved by rest. The patient described the pain as a deep aching, localized, nonradiating sensation. The patient had a history of right anterior hip pain since 2015 that occurred during exercise, most notably due to running and kicking. Recently, in the month preceding his visit, the patient developed acute right anterior-middle thigh pain, which increased in severity after playing kickball. He described a bothersome and palpable lump in the right anterior-middle thigh, which interferes with his daily activities and exercise regimen. He also noted intermittent right hip pain focally at the anterior iliac crest region with a clicking sound during hip flexion and extension. He did not report any radicular symptoms, lateral hip trochanteric pain, or loss of hip joint range of motion (ROM).

During the physical exam, tenderness was noted near the anterior superior iliac spine (ASIS) and AIIS along with a well-circumscribed mobile mass in the mid-anterior thigh. Differential diagnosis included a potential RF tear, but the additional proximal symptoms suggested indirect or direct RF avulsion. A snapping hip syndrome was also considered, given the popping during hip ROM. 

**Figure 1:**
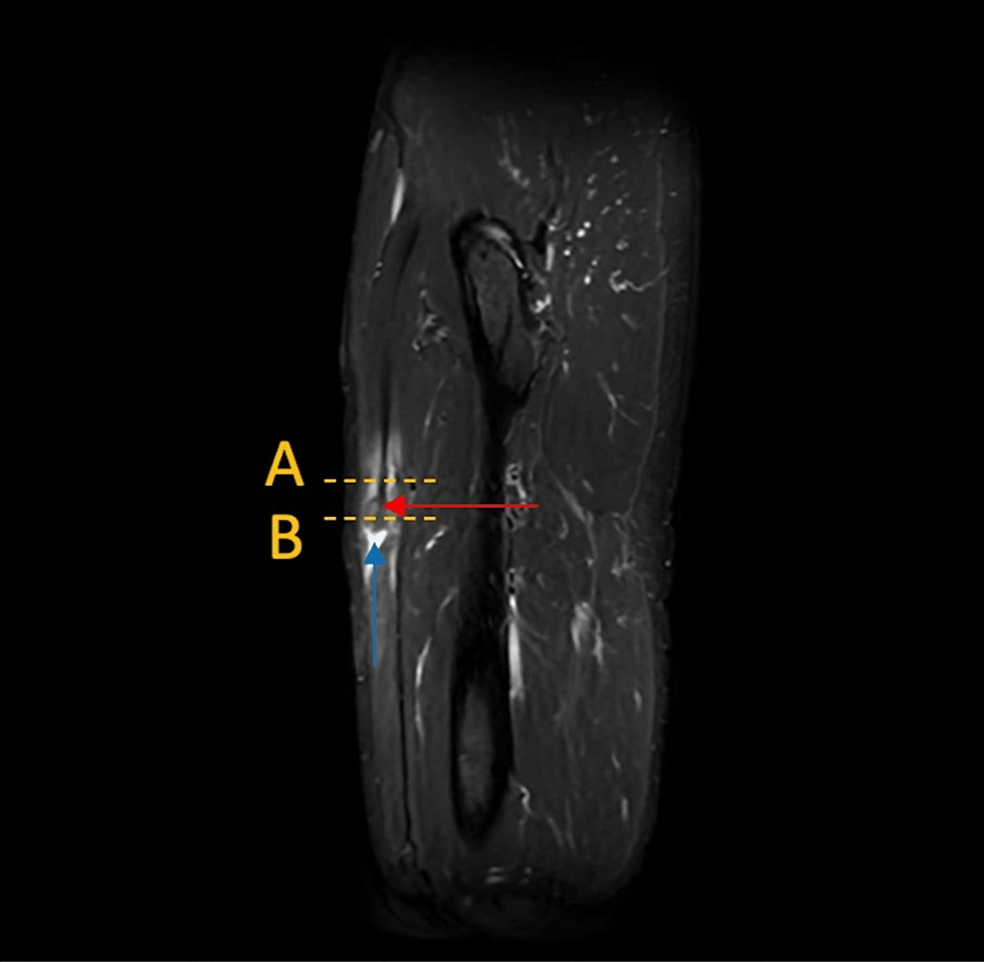
Sagittal STIR MRI demonstrating the combination of tearing (blue arrow) and up to 23 mm retraction of the inner muscle (red arrow). This is analogous to a finger in a glove, also known as an intramuscular degloving of the rectus femoris. STIR, Short Tau Inversion Recovery; MRI, magnetic resonance imaging

**Figure 2:**
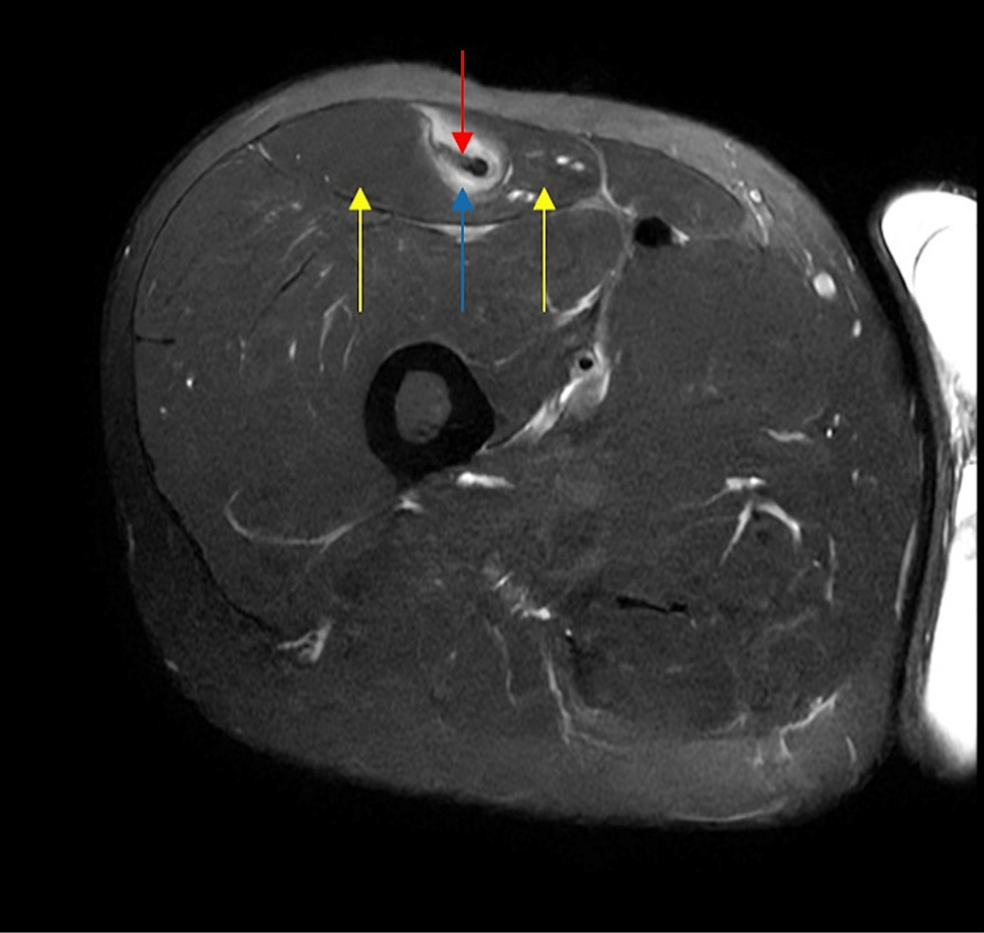
Axial T2 fat-suppressed MRI through the mid-thigh demonstrating an edematous and torn inner bipennate intramuscular portion (red arrow), the finger, with separation and a small amount of intervening fluid and likely blood products (blue arrow) from the surrounding outer unipennate portion of the muscle (yellow arrows), the glove, which appears normal. MRI, magnetic resonance imaging

The patient's previous hip pain did not improve with conservative management (i.e., rest, activity modification, adjustments to a home exercise program, and two rounds of physical therapy starting in 2015 and again within the past three months). Out of suspicion of a potential RF tear prompted by the physical exam and the patient’s acute presentation, advanced imaging was clinically indicated, and an MRI was ordered to further assess the RF region and its origin. Right-thigh MRI without contrast (Figure 1) revealed an IDI of the RF muscle, with edema within the inner and outer muscular portions of the RF tear region and the so-called *finger-in-the-glove* sign. There was a 23 mm retraction of the torn inner indirect myotendinous complex (myotendinous junction, or MTJ) of the RF (Figure 2). The MRI findings, particularly the presence of edema, were indicative of a current injury instead of the hip pain from 2015. Hence, the hip pain mentioned by the patient was unlikely related to the current presentation and the IDI is the true underlying cause. After consulting with orthopedic surgery, the patient did not require surgery or other intervention, including injection (such as a platelet-rich plasma injection) for the IDI. He was advised to avoid aggressive quadriceps contractions and high-intensity, high-impact exercise. It was suspected he may have had snapping hip syndrome as well, but the IDI was the main contributor to symptoms based on the MRI. He also initiated physical therapy, and after six to eight weeks, he experienced an improvement in ROM and strength, along with improved tolerance to physical activity and exercise. At a one-year check-in via electronic health record, the patient did not yet make a full recovery but reported improvement in mobility and is now able to run occasionally.

## Discussion

While injuries to the RF are common, intramuscular degloving is an uncommon and seldom reported form of RF injury. Only 10 reported cases exist in the literature, with eight of those cases coming from the same source (Table 1). Additionally, 50% of the reported intramuscular degloving cases were initially misdiagnosed, leading to prolonged recovery time and delayed return to sport (Table 1). Previous reports have identified kicking sports as being particularly high risk for intramuscular degloving of the RF [11], and our case is the first to describe intramuscular degloving in a kicking sport besides soccer. Kicking an inflated ball resulting in substantial deformation of the ball itself is thought to contribute to increased forces acting on the muscles involved in kicking [13-14]. Past research focuses on this phenomenon in soccer, and further investigation should be done to identify how the unique properties of a kickball ball potentially exacerbate this effect. From an anatomical perspective, as the RF is a biarticular muscle crossing two joints, it may be at additional unique risk resulting in opposing pulling forces causing a degloving event [13]. Related, eccentric contractions (which can occur in kicking, but also during sprinting such as from home to first base) also may create similar opposing muscle-pulling forces [15].

**Table 1:** Comparison of previous case physical exam findings, imaging, and diagnoses.

Case(s)	Case reports	Physical exam findings	Imaging (reported in the order received)	Diagnosis
1	Mallick and Anshul [7]	Diffuse tenderness and palpable swelling over the mid-thigh about 12 cm in length. Thigh girth increased by 1 cm. Inability to perform active straight leg raise. Painful resisted knee extension, active prone knee flexion, and stretching of quadriceps. Reduced strength in right hip flexion and right quadriceps as measured by a digital muscle dynamometer. Positive Ely’s test.	X-ray: No abnormalities. MRI: The presence of fluid separating the outer and inner rectus fibers extending over 5-6 cm with proximal retraction of the inner fibers along the central tendon	Intramuscular degloving injury of the rectus femoris
2	Krebs et al. [16]	Tenderness over the anterior mid-proximal thigh about 10 cm in length. Tenderness is worse medially than laterally with no palpable muscle defect. Full active and passive range of motion and strength of hip and knee. Positive fulcrum and hop test.	X-ray: No abnormalities. MRI: Acute complete disruption of the inner bipennate component of the RF from the superficial unipennate component with retraction of the inner component.	Closed degloving injury of the rectus femoris

From a clinical standpoint, other aspects of kickball may contribute to RF injuries. Athletes have substantial periods of downtime with minimal movement while their team is fielding and while waiting in line to go up to *bat*, or kick [17]. After these periods of immobility, athletes are immediately tasked with powerful kicks to the ball, often without adequate warm-up. This transition from immobility to immediate substantial effort risks injury to the kicking muscles, including the RF [18]. Unfortunately, the literature on the epidemiology of kickball injuries is extremely sparse, as it is largely considered a casual/recreational activity. However, past studies have suggested that kickball is a popular game with an underappreciated injury burden [19].

Finally, all previously reported cases of IDI in the RF occurred in young, competitive athletes (aged 15-22 years), indicating that this group may be at particularly high risk. However, it is not known if this is due to the unique demands of higher-level sports or underreporting among athletes in less competitive sports or athletes who are older. Our identification of an older (38 years) individual injured while playing recreational kickball suggests that the prevalence of the injury may be broader than previously thought. Recreational or social athletes can be at higher risk of injury due to not performing warm-ups, performing sports techniques without the supervision of a sports coach, or not performing injury-preventive activities [20].

Previous literature has also pointed out that while IDIs are serious and debilitating, they are underreported and often unrecognized, which can lead to delays in appropriate treatment, extended workup times, and delayed returns to play [21]. Specifically, while surgery was not indicated in any of the reported cases of intramuscular degloving to the RF, other degloving injuries have been shown to require surgical intervention [21]. As a result, failure to quickly identify intramuscular degloving of the RF requiring surgical intervention could negatively impact recovery. Our case highlights the need to increase provider awareness and lower the bar of suspicion for adequately assessing for IDI in the RF.

Epidemiology

While the prevalence of injuries to the RF, in general, is high, the true rate of IDIs is difficult to establish due to a paucity of epidemiological data and may be underreported. Previously, degloving injuries have been estimated to represent only 9% of all RF injuries [7]. This estimate was based on limited sample size and time frame. Due to the high prevalence of RF injuries in sports as well as the absence of IDIs in standard muscle injury classifications [22], it is likely that the prevalence of IDIs to the RF is underestimated. This is further complicated by the fact that the symptoms of IDIs in the RF are broad and may overlap with other quadriceps injuries. In addition, diagnostic confirmation of an IDI essentially requires an MRI, which can be cost-prohibitive and time-consuming to schedule. This can lead to delays in diagnosis and treatment in some cases [23]. These factors may lead to further underreporting. However, the high prevalence of quadriceps injuries in kicking sports, especially in the RF, suggests that the prevalence of IDI in the RF is substantial. Future investigations should focus on determining the true prevalence of IDI in the RF.

Anatomy

The RF is particularly susceptible to injury due to its unique attributes, such as crossing two joints and having a high percentage of type II fibers (approximately 65%) [24]. Additional RF characteristics that may contribute to injury include the muscle’s double tendinous origin (indirect and direct heads) with the indirect tendon having a long intramuscular extension (also referred to as intramuscular septum or central aponeurosis). This anatomy of interlayered muscle and tendon creates a *muscle within a muscle* configuration [24]. The inner component has a bipennate structure, which is surrounded by unipennate muscle. The outer unipennate muscle contains fibers, all of which originate from the same side of the tendon. The bipennate muscle consists of a central tendon with fibers originating on both sides. This *muscle within a muscle* anatomy differentiates the RF from other quadriceps muscles and makes the RF particularly susceptible to intramuscular degloving as this mechanism of injury involves the separation of an inner layer from an outer layer [8].

Mechanism of injury

Existing literature points to kicking as the most common mechanism of injury causing intramuscular degloving in the RF [6]. Sprinting has also been identified as an alternative mechanism [7]. Both sprinting and kicking involve eccentric muscle action, known to be the most common cause of muscle strain and tear [4]. In addition, both actions significantly load the quadriceps muscles, especially the RF [4], which makes such activities particularly conducive to an intramuscular degloving event. Degloving injuries often occur at the MTJ. The MTJ is responsible for transmitting force generated in muscles to their associated tendons, a function particularly important in high-intensity and high-impact activities such as kicking and sprinting [4,25].

While mechanistic details of intramuscular degloving have not been rigorously established, the hypothesized mechanism is thought to be a shearing phenomenon resulting from outer unipennate fibers and inner bipennate fibers acting independently [1], causing dissociation of the inner muscle belly from the outer muscle belly. Such shearing forces are unique to the muscle within a muscle anatomy of the RF. As this type of injury is thought to contribute to longer rehabilitation times associated with central tendon injuries [26], the index of suspicion for an IDI should be high to avoid delays in diagnosis and prolonged recovery.

Risk factors

As discussed above, specific risk factors for IDI in the RF depend on the particular activity at the time of injury. In sprinting, the risk for injury may be elevated during acceleration and deceleration [4], and it is likely increased during eccentric contractions. History of previous injuries to the RF also can increase risk [7,27].

While increased age is often considered a risk factor for injury, it has not previously been shown to be a risk factor for quadriceps muscle injury [4]. Leg dominance, however, may be a risk factor. In a case series of soccer players, more IDIs occurred in the dominant leg than the nondominant leg [6], which aligns with injuries to quadriceps muscles in general [28-29]. A paucity of reported cases of IDI makes it difficult to establish a comprehensive list of risk factors for IDI, but other risk factors pertinent to a quadriceps injury, in general, include lack of flexibility, poor strength, short height, and increased body weight [4,29].

Diagnostic workup

The clinical presentation of IDI in the RF can vary. Special attention should be given to the activity at the time of injury. If the individual was engaged in kicking, sprinting, or similar activity that loads the RF, IDI should be included in the differential diagnosis (Table 1). Some patient reports of the history include tenderness and swelling over the mid-thigh region (Table 2), consistent with this case. However, the symptoms can be quite nonspecific. On physical exam, while some cases have shown no palpable muscle deformity [16], our patient presented with a well-circumscribed mobile mass in the thigh. Additionally, while previous physical exam findings focused on the mid-thigh region (Table 2), our patient also had symptoms at the RF origin, suggesting additional pathology (indirect or direct RF strain) and/or concurrent hip flexor pathology (such as snapping hip syndrome). Therefore, diagnosing IDI from history and physical exam alone may be difficult, warranting the need for imaging [30].

**Table 2:** Review of published cases of intramuscular degloving injury. NSAIDs, non-steroidal anti-inflammatory drugs; PNF, proprioceptive neuromuscular facilitation

Case(s)	Case reports	Age/gender	Sport affiliation	Activity at the time of injury	Symptoms	Initial misdiagnosis (Yes/No)	Treatment	Return to play interval
1	Mallick and Anshul [7]	22/Male	Track and field	Sprinting	Dull, diffuse pain over the mid-thigh region. Nonradiating and aggravated by stretching, running, and sprinting	No	Activity modification, ice, NSAIDs, isometrics, active-assisted range-of-motion exercises, and passive and PNF stretching of quadriceps.	84 days
2	Krebs et al. [16]	21/Female	Lacrosse	Unknown	Soreness during practice progressing to throbbing after games. Pain waking the patient from sleep and pain exacerbated by leg hanging over a chair.	Yes (quadriceps strain)	Activity modification, rehabilitation therapy in training room, NSAIDS, ice, cupping, needling - rest, range-of-motion exercises, isometrics, dynamic exercises, and lacrosse-specific drills	Estimated 56 days
3-10	Kassarjian et al. [6]	15-22/Male	Soccer	4/8, kicking; 1/8, sprinting; 1/8, forced quadriceps extension; 2/8, unreported	Unreported	4/8 Yes (overuse injury)	Unreported	Average 38.7 days (range of 28-58 days)

The modality of choice is MRI [21]. MRI intensity and enhancement patterns vary based on the age, size, shape, and degree of fluid accumulation within the injury. Information regarding the fluid collection is particularly useful in determining how the injury will be managed (i.e., needle aspiration, operative incision, and drainage). Other diagnostic techniques, if a diagnosis of degloving injury is suspected, include CT (not often required) and potentially ultrasound; the current utility of ultrasound is still questionable and warrants further investigation [30].

Differential diagnosis

In our patient, a strain of the RF, a partial or complete RF tear, snapping hip syndrome, and an ASIS/AIIS avulsion fracture were considered potential diagnoses. Symptom overlap can exist between these injuries and IDIs, including localized edema, ecchymoses, tenderness, pain with passive and active ROM, and pain with weight bearing on the affected leg [31-32]. Other conditions that may mimic degloving injuries include seroma, abscess, compartment syndrome, and deep vein thrombosis [8]. A thorough patient history and physical examination, with appropriate imaging as indicated, can often rule out these alternative diagnoses and reveal a degloving injury, as was the case in our patient. For example, the absence of trauma may point against a fracture, seroma, or abscess; a lack of response to conservative management suggests a more severe injury than an RF strain. 

Treatment/Management

There is no established guideline or consensus on the management of degloving injuries [21]. This may be because degloving injuries do not fit into the traditional three-level classification system for muscle injuries. Furthermore, because of the scarcity of literature, treatment recommendations provided herein are largely based on available clinical recommendations, expert opinion, and case series data. Initial management of degloving injuries is largely dependent on the amount of subdermal fluid collection, which can lead to tissue necrosis if large amounts are allowed to accumulate. This is most important in the initial presentation; however, it has less of a role if there is a delay in the patient presenting for medical care. To address this, rest, compressive wraps, and needle aspiration can be utilized; sclerosing agents (i.e., doxycycline, erythromycin, and vancomycin) and surgical irrigation and debridement can be employed in more extreme cases if needed. Typically, conservative measures, including rest and compressive therapy, are sufficient to treat most cases of mild-to-moderate IDI. If there is a significant fluid accumulation that warrants aspiration to reduce symptoms, and more than 50 mL of fluid is removed, operative interventions are recommended [33]. The standard of care, as suggested by Mayo Clinic guidelines, in this case, is incision and drainage [33]. Incision size and whether the wound remains open or closed after debridement are based on the specific wound, patient, and surgeon. Negative pressure dressing has been shown to aid in wound closure [34]. Currently, there is a lack of sufficient literature on the use of other measures, including chiropractic or acupuncture, in the treatment of IDI, and further research is indicated. 

Prognosis and complications

Optimal outcomes for patients with degloving injuries are strongly associated with early presentation and prompt identification [21]. If quickly recognized and managed appropriately, with proper treatment adherence to conservative measures and interventions, complete resolution of symptoms is often obtained. 

Recurrence is the most common complication, estimated at approximately 56% of patients who had large fluid accumulation requiring aspiration versus 19% in patients who received conservative measures such as rest and compression therapy [8]. This demonstrates an association between the severity of injury and recurrence rate, as more severe injuries require aspiration as opposed to compression therapy. Other complications include skin necrosis, bacterial infection, and in extreme cases necrotizing fasciitis [21]. When considering these factors, it becomes clear that early recognition of injury and prompt management can reduce the risk of complications and recurrence. 

Prevention and patient education

Given that intramuscular devolving injuries are not well reported, it is understandable that many patients may not be familiar with this type of injury. Patients most susceptible to this injury (i.e., those who participate in high-intensity and high-impact physical activity, especially kicking and sprinting sports) should be educated on the possibility of this injury. Understanding the risks may enable these patients to seek earlier medical attention. Potential prevention strategies may include cross-training and strengthening the RF. Patients may consider adding eccentric exercise progressions into their workouts. The MTJ is exposed to the highest loads during eccentric contractions, making it most vulnerable to a degloving injury during this phase. Increased eccentric muscle strength is protective against muscle strains in general, so it may have similar benefits for preventing degloving injuries [25]. Additionally, other potential prevention strategies, such as flexibility training and proper warm-up and cool-down routines, may be recommended, as limited warm-up has been demonstrated to contribute to an increased risk of an IDI [18]

Enhancing healthcare team outcomes

A patient presenting with a degloving injury will interact with a variety of healthcare professionals. If the injury was brought on by acute trauma, emergency room physicians and nurses will likely be the first to assess the patient. If a patient did not have acute trauma or perhaps did not recall a single major inciting event, a primary care or sports medicine provider may be the first to encounter this condition. Orthopedists and physicians with sports-medicine expertise are likely to become involved in care, as well as physical therapists and ancillary support staff such as massage therapists and acupuncturists. A delayed diagnosis and/or misdiagnosis of this injury not only has physical implications for the patient (i.e., prolonged pain, increased risk of complications and infections, and increased likelihood of incomplete recovery) but also potential emotional and psychosocial consequences (i.e., stress from uncertainty about injury and inability to participate in enjoyed physical activities). Additionally, the patient may experience an extra financial burden (i.e., having to pay for more appointments, imaging, and procedures). Increasing awareness of intramuscular degloving injuries among healthcare professionals should improve the outcomes of patients by facilitating prompt diagnosis and timely management of the injury.

## Conclusions

The RF muscle is characterized by a unique muscle-within-a-muscle structure. The unique anatomy of the RF, combined with its biarticular nature and involvement in eccentric contractions during high-intensity sports such as kicking and sprinting, makes it susceptible to injury. IDIs are underrecognized and not typically included in traditional muscle injury classifications. Herein, we describe a 38-year-old male with an IDI of the RF involved in the sport of kickball. Given the high prevalence of RF muscle injuries in sports and its unique anatomy, providers should be aware of this unique injury. Kicking sports, in general, may increase risk (such as ball sports and martial arts), although kickball may be unique due to the additional elastic loading from increased deformation of the kickball compared to other ball sports, resulting in stronger opposing muscle forces within this unique anatomy. Our case highlights the need for more research to identify unique mechanisms and multifaceted risk factors that predispose athletes to such injuries. Factors such as fatigue, overexercising, and strenuous physical exertion could potentially increase the risk of IDIs by exacerbating mechanical stress on the RF muscle. An improved understanding of these factors will inform the development of preventative strategies and contribute to better clinical outcomes for patients who suffer IDIs in the future.
